# Joint B Vitamin Intake and Type 2 Diabetes Risk: The Mediating Role of Inflammation in a Prospective Shanghai Cohort

**DOI:** 10.3390/nu16121901

**Published:** 2024-06-16

**Authors:** Yang Zhu, Tao Ying, Mingjing Xu, Qing Chen, Min Wu, Yuwei Liu, Gengsheng He

**Affiliations:** Key Laboratory of Public Health Safety of the Ministry of Education, School of Public Health, Fudan University, Shanghai 200032, China; 21211020175@m.fudan.edu.cn (Y.Z.);

**Keywords:** type 2 diabetes, B vitamins, joint exposure, inflammation, mediation

## Abstract

Background and Aims: Type 2 diabetes (T2D) is a global and complex public health challenge, and dietary management is acknowledged as critical in its prevention. Recent studies have highlighted the involvement of micronutrients in T2D pathophysiology; our study aims to assess the association between B vitamin intake and T2D risks and the mediating role of inflammation. Methods: In a prospective cohort design, data on B vitamins intake, including thiamine (B1), riboflavin (B2), niacin (B3), pyridoxine (B6), folate (B9), and cobalamin (B12), was obtained using a validated food frequency questionnaire (FFQ), and blood inflammatory biomarkers were analyzed according to standard protocol in the local hospitals at baseline from 44,960 adults in the Shanghai Suburban Adult Cohort and Biobank (SSACB). Incident T2D cases were identified according to a physician’s diagnosis or medication records from the electronic medical information system. We employed logistic and weighted quantile sum regression models to explore the associations of single and combined levels of B vitamins with T2D and mediation analyses to investigate the effects of inflammation. Results: Negative correlations between B vitamins and T2D were observed in the single-exposure models, except for B3. The analyses of joint exposure (B1, B2, B6, B9, and B12) also showed an inverse association (OR 0.80, 95% CI 0.71 to 0.88), with vitamin B6 accounting for 45.58% of the effects. Further mediation analysis indicated a mediating inflammatory impact, accounting for 6.72% of the relationship. Conclusions: Dietary intake of B vitamins (B1, B2, B6, B9, B12) was associated with a reduced T2D risk partially mediated by inflammation in Shanghai residents.

## 1. Introduction

Diabetes has become an increasingly critical public health challenge worldwide. According to the International Diabetes Federation Diabetes Atlas (2021), 537 million adults (10.5%) aged 20–79 have diabetes, with the majority residing in low- to middle-income countries. In China, the prevalence of diabetes in 2018 exceeded 12%, surpassing the global average, and this number continues to rise [1]. The World Health Organization predicts that by 2030, diabetes will become a leading cause of death globally [2]. Diabetes mellitus encompasses two main types: type 1 diabetes (T1D) and type 2 diabetes (T2D). T1D is characterized by the autoimmune destruction of insulin-producing beta cells in the pancreas, leading to an absolute insulin deficiency. In contrast, T2D, which accounts for approximately 90–95% of diabetes cases, is primarily defined by insulin resistance and relative insulin deficiency. Insulin resistance is a condition where the body’s cells do not respond effectively to insulin, which is crucial in the pathogenesis of T2D.

Mounting evidence suggests that DNA methylation plays a pivotal role in developing metabolic disorders, including T2D and its associated complications [3,4]. DNA methylation can be influenced by environmental factors such as diet [5]. These dietary factors include consuming methyl-donor nutrients involved in one-carbon metabolism, such as B vitamins (B2, B6, B9, and B12). The intake of these nutrients could potentially affect the T2D risks through epigenetic alterations [6]. Currently, there is a lack of research investigating the overall association between methyl-donor nutrients and the risk of T2D [7]. Moreover, there is limited information regarding the contributions of individual B vitamin intake to the combined effects on T2D risks.

B vitamins are essential micronutrients for numerous metabolic processes vital for human health. They act as co-factors for various enzymes involved in energy metabolism, protein synthesis, and other functions [8]. However, the regulatory mechanisms of B vitamins on T2D largely remain undiscovered. While the precise causes of insulin resistance, a primary pathological mechanism of T2D, are not entirely understood [9,10,11], it may be attributed to various biological processes, including oxidative stress and inflammatory responses. For instance, several studies have shown that inflammatory markers are involved in the insulin actions in the target tissues and the development of insulin resistance [12]. Meanwhile, a randomized controlled clinical trial has suggested that high-dose B vitamin supplements reduce oxidative stress and inflammation [13]. Studies have shown that adding vitamin B1 to macrophages could inhibit oxidative stress and the release of pro-inflammatory cytokines [14]. Vitamin B2 can inhibit the migration of neutrophils and the infiltration and accumulation of activated granulocytes in peripheral areas, potentially reducing inflammatory responses [15]. In plasma, a derivative of vitamin B6, pyridoxal 5’-phosphate (PLP), is negatively correlated with inflammatory biomarkers [16]. High-dose oral folic acid could reduce inflammation in mice by inhibiting the secretion of T-cell cytokines [15]. B vitamins are also involved in one-carbon metabolism (B2, B6, B9, B12) to participate in the methylation of homocysteine to methionine. This methylation can prevent the accumulation of homocysteine, impacting DNA synthesis and repair, consequently reducing oxidative stress and inflammatory responses [15].

A cross-sectional study has illuminated the beneficial impact of vitamin B1 on T2D [17]. Notably, a significant U-shaped correlation was observed in a Chinese cohort study [18], with the optimal range identified between 0.75–1.1 mg/day. This has been further corroborated by meta-analyses concluding the efficacy of vitamin B1 in reducing T2D risk [19]. Meanwhile, the role of vitamin B2 has been characterized by its inverse correlation with T2D, hinting at a protective role against this disease [20]. However, the studies regarding vitamin B3 demonstrate a more contradictory picture. While cross-sectional studies have indicated an inverse correlation [20,21,22], Pan et al. have reported a positive correlation, underscoring the complexity of this relationship [23]. Folic acid (vitamin B9) presents another intriguing case. A cross-sectional study has identified an inverse U-shaped relationship with T2D [21], while a cohort study in Chinese populations links low folic acid levels with increased T2D risk [24]. Some meta-analyses have shown folic acid’s potential to lower fasting blood glucose, with another meta-study finding no significant correlation [25]. Furthermore, a study investigating the association between mixed B vitamins and T2D observed an inverse correlation; however, it was non-statistically significant, which might be attributed to the limited sample size and the constraints of the cross-sectional study design [26].

In summary, given the mechanistic links between B vitamins and inflammation and T2D, we hypothesize that the intake of B vitamins is inversely associated with the risk of developing T2D and that this association is mediated by a reduction in inflammatory responses, subsequently lowering the risk of T2D.

## 2. Research Design and Methods

**Figure 1:**
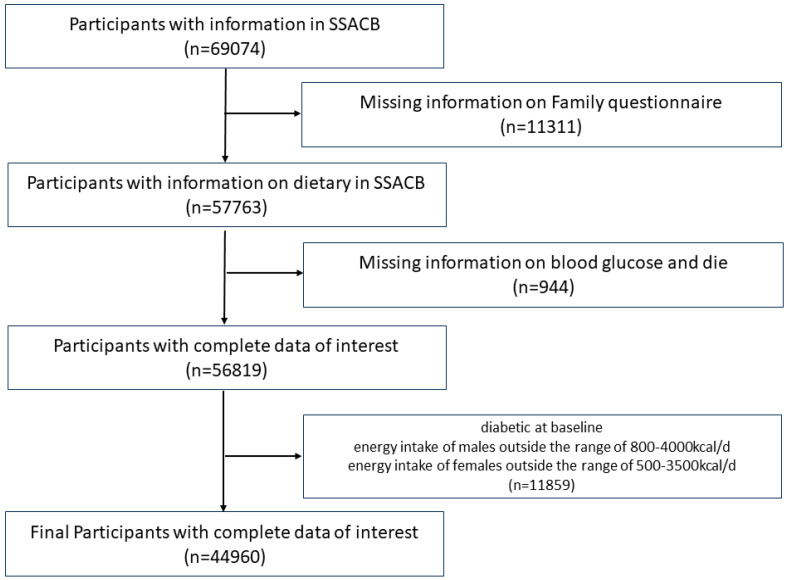
Flow chart of participant selection. SSACB, The Shanghai Suburban Adult Cohort and Biobank.

The Shanghai Suburban Adult Cohort and Biobank (SSACB) is an ongoing cohort study to assess the public health development of adult populations in Shanghai, China. At baseline, face-to-face interviews were conducted to collect data on demographics, diets, lifestyle, and general health conditions. Subsequent physical examinations were performed, and blood and urine samples were collected. Moreover, the cohort incorporated a health information system comprising electronic medical records, a chronic disease management system, a cancer registry, an infectious disease reporting system, and a death registration system to gather information on the incidence of T2D. All protocols and implementation methods can be found in our previous reports [27]. We set the cutoff date for follow-up as 1 March 2022. We excluded individuals with missing vital records and those who died during the study or who already had diabetes at baseline. Subsequently, a screening based on daily energy intake (male: 800–4200 kcal/d, female: 500–3500 kcal/d) was conducted, resulting in the final inclusion of 44,960 participants in the study (Figure 1).

### 2.1. Study Populations

#### 2.1.1. Ascertainment of Incident T2D Cases

In this study, the T2D status at baseline was determined through a combination of self-reported questionnaires, blood tests, and medical system records. To eliminate all the individuals with pre-existing diabetes (type 1 and 2) at baseline, based on the Chinese diagnostic criteria for diabetes, we excluded self-reported diabetes, individuals with glycated hemoglobin levels greater than 6.5%, and diabetes-related medical records before enrollment. To determine T2D incidence, all participants’ T2D-related medical diagnostic information and medication history were utilized to assess the occurrence of type 1 diabetes or T2D and the onset time of the condition after enrollment. The physicians strictly adhered to the 2013 edition of the “Guidelines for the Prevention and Treatment of Type 2 Diabetes in China” when diagnosing and categorizing the types of diabetes. The antidiabetic medications utilized include insulin, sulfonylureas, insulin secretagogues, insulin sensitizers, incretins-related agents, and α-glucosidase inhibitors. The time when a patient was first diagnosed with diabetes or first started using related antidiabetic medications was defined as the onset time of their disease.

#### 2.1.2. Measurement of Dietary B Vitamins Sources

Face-to-face interviews on diets were conducted by trained investigators using a validated food frequency questionnaire (FFQ) at baseline to assess the intake of nutrients from food groups, while a household questionnaire (HQ) was used to evaluate the nutrient intake from seasoning, including cooking oil, sugar, salt, and soy sauce. The sum of FFQ and HQ represented the overall intake of nutrients at an individual level. The validity of the FFQ has been evaluated [28]. The FFQ was designed using food groups, incorporating a total of 29 food categories. The consumption frequency for these food groups was set across eight levels: never; less than once a month; 1–3 times a month; 1–3 times a week; 4–6 times a week; once a day; twice a day; three or more times a day. These dietary frequency levels were converted into daily intake frequency scores of 0, 0.02, 0.07, 0.29, 0.71, 1, 2, and 3, respectively. The average nutrient content of the food groups was calculated using weighted averages based on the sixth edition of the China Food Composition Database, and the average food consumption in Shanghai. The latter consumption data was derived from the Shanghai Food Consumption Survey (SHFCS) conducted by Fudan University from September 2012 to August 2014 [29], which used the 24-h dietary recall method (once per season) to measure dietary intake across different populations over four occasions. Regarding B vitamins, we calculated the individual intake levels of thiamine (B1), riboflavin (B2), niacin (B3), pyridoxine (B6), folate (B9), and cobalamin (B12).

#### 2.1.3. Measurement of Blood Inflammatory Biomarkers

A total of 16 mL of fasting blood was drawn into four vacuum blood collection tubes for blood samples. From these, 2 mL into a tube containing EDTA and 4 mL into a serum separator tube were sent immediately for clinical laboratory testing. The remaining 10 mL of blood was centrifuged to separate the serum and red blood cells, with the separated samples stored at −80 °C for future use or biobank purposes. High-pressure liquid chromatography (TOSOH G8, Automatic Hemoglobin A1c Analyzer, Tosoh Corporation, Tokyo, Japan) was used to measure glycated hemoglobin (HbA1c). The Roche COBASC501 automatic biochemical analyzer (Roche Diagnostics, Basel, Switzerland) was utilized to quantify white blood cell count, monocyte percentage, monocyte count, lymphocyte percentage, lymphocyte count, urine creatinine, basophil percentage, basophil count, aspartate aminotransferase (AST), alanine aminotransferase (ALT), eosinophil percentage, eosinophil count, serum creatinine, neutrophil percentage, neutrophil count, serum albumin, and serum globulin.

#### 2.1.4. Covariates

Covariates were identified based on previous literature [30]. All covariates were sourced from face-to-face interviews and physical examinations, including age and sex (male or female), body mass index (BMI), glycated hemoglobin (HbA1c), family history of diabetes (yes or no), total energy intake from FFQ survey, smoking (yes or never), drinking (yes or never), an education level (primary school or below, junior high school, or senior high school or above), and physical activity (low, medium, or high). Physical activity assessment was conducted using the International Physical Activity Questionnaire (IPAQ). According to the truncation principle, physical activity durations were truncated twice. The first truncation occurred when the average daily duration of any physical activity exceeded 3 h, in which case it was recoded to 180 min. The second truncation occurred when the weekly duration of physical activity at any intensity level exceeded 1260 min, subsequently recoding the weekly duration of that intensity level to 1260 min. Physical activity levels were classified into low, moderate, and high categories based on MET values and activity durations, following the IPAQ Working Group’s recommendations [31].

### 2.2. Statistical Analyses

All analyses were performed with R (version 4.2.3). Chi-square tests or Fisher’s exact tests for categorical variables and t-tests or Mann–Whitney U tests for continuous variables were used to assess participants’ demographic characteristics by T2D status. The levels of B vitamins were categorized into four quantiles (Q1, Q2, Q3, Q4) as categorical variables. Multivariable logistic regression was applied to estimate odds ratios (OR) and corresponding 95% confidence interval (CI) for the associations of single B vitamin and incident T2D risk. The trend test across increasing exposure groups was calculated using integer values (1, 2, 3, and 4).

Weighted quantile sum regression (WQS) was applied to explore the overall effects of B vitamins on T2D and the weight estimate of each B vitamin. R package (“gWQS”) calculated the WQS index comprising weighted sums of individual relevant B vitamin intake, representing the mixed intake levels of B vitamins, and the components of concern were identified by weights. The final result could be interpreted as the concurrent impact on T2D of a one-quantile increase of mixed B vitamins.

Given the potential nonlinear and non-additive dose-response relationships among B vitamin exposures, we matched one T2D case to two controls for Bayesian Kernel Machine Regression (BKMR) analysis. We utilized the “bkmr” package in R software to evaluate the joint effects of combined B vitamin exposures on the risk of T2D. The exposure-response and dose-response functions were also obtained for individual B vitamin intake and T2D risks to estimate interactions between single B vitamins when holding other B vitamins fixed.

Sensitivity analyses were also performed using the Quantitative G-computation (QGComp) method to eliminate the amplification bias caused by WQS regression when the relevant directions are not pre-considered. This approach combines WQS regression and G-computation without assuming a direction homogeneity [32]. We utilized the “qgcomp” R package to obtain positive and negative weight coefficients for each component in the B vitamin mixture.

The potential mediating effects on the associations of mixed B vitamins (shown as WQS index) with T2D risk were evaluated by mediation models (R package “CMAverse”), using the baseline blood inflammation biomarkers as mediators. We then incorporated the relevant biological markers into a joint mediation model to calculate the overall mediating effect. The direct effect (DE) represented the effects of B vitamin intake on diabetes without a mediator. The indirect effect (IE) described the effects of B vitamins on T2D through the biological markers. The proportion of mediation (PM) was calculated by using IE divided by TE (DE + IE). Statistical significance was set at *p*-value < 0.05.

## 3. Results

### 3.1. Population Characteristics and B Vitamin Intakes

In the SSACB, after a median follow-up period of 4.65 years, among the 44,900 participants enrolled at baseline, 1839 incident cases of T2D were recorded, and detailed baseline characteristics and B vitamin intake are presented in Table 1. Overall, significant differences in age, BMI, blood HbA1c, family history of diabetes, drinking alcohol, physical activity, educational level, and marital status were observed at baseline between individuals who developed T2D and those who did not (*p*-value < 0.05). Individuals with newly diagnosed diabetes were more likely to be older, have a higher BMI, and consume alcohol. The intake of B vitamins, except for B9, were significantly lower in the participants with T2D than those without T2D (*p*-value < 0.05).

### 3.2. Contribution of Food Groups to B Vitamin Intake among Participants

The average contribution of 34 food groups to the intake of B vitamins among the study participants is presented in Table 2. Rice and its products were the highest contributors to vitamins B1, B3, and B6, contributing 36.02%, 32.17%, and 27.52%. Fresh vegetables had the highest contribution to vitamins B2 and B9, contributing 14.58% and 29.04%. Shrimp, crab, and other mollusks were the top contributors to vitamin B12, contributing 24.37%.

### 3.3. Associations of Single B Vitamin Intake with Risk of Incident T2D

**Figure 2:**
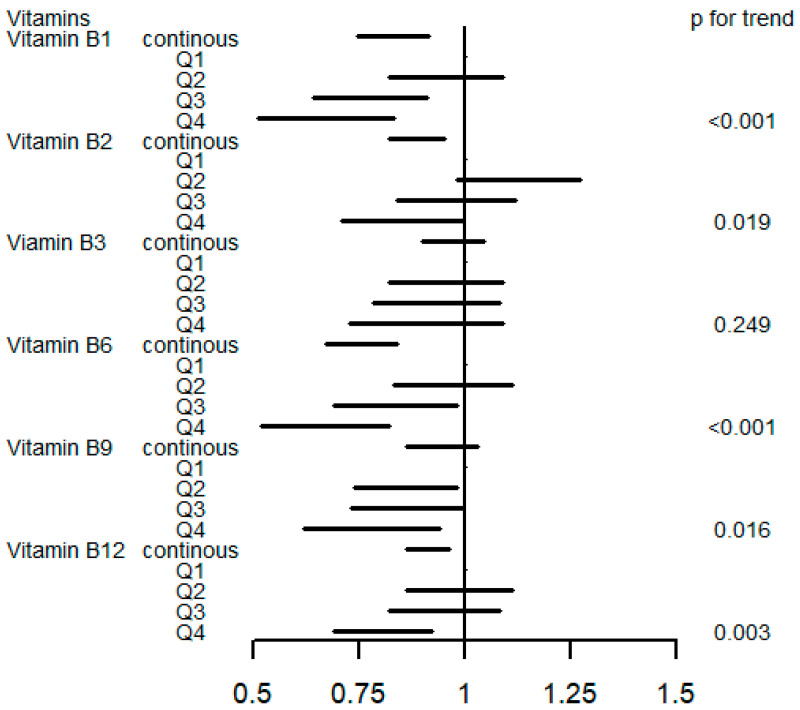
OR (95% CI) in T2D associated with single B vitamin levels. Models were adjusted for sex, age, HbA1C (%), education, smoking status, drinking alcohol status, physical activity, body mass index, energy intake, and family history of diabetes. Q, quartile.

Figure 2 and Table S1 display the association between a single B vitamin intake and the risk of incident T2D by the logistic regression models. As continuous variables, dietary vitamin B1 (OR 0.82, 95%CI 0.74 to 0.91), vitamin B2 (OR 0.88, 95%CI 0.82 to 0.95), vitamin B6 (OR 0.75, 95%CI 0.67 to 0.84), and vitamin B12 (OR 0.91, 95%CI 0.86 to 0.96) were associated with a reduced risk of incident T2D. The highest intake of vitamin B1 (OR 0.65, 95%CI 0.51 to 0.83), vitamin B2 (OR 0.84, 95%CI 0.71 to 0.99), vitamin B6 (OR 0.65, 95%CI 0.52 to 0.82), vitamin B9 (OR 0.77, 95%CI 0.62 to 0.94), and vitamin B12 (OR 0.80, 95%CI 0.69 to 0.92) decreased the risk of diabetes compared to the lowest quantile (*p* for trend <0.05). Table S2 presents the logistic model results without adjusting baseline glycated hemoglobin; the associations between incident T2D and vitamin B1, B6, B9, and B12 remained statistically significant (*p*-value < 0.05).

### 3.4. Association of Joint B Vitamin Intake with Risk of Incident T2D

**Figure 3:**
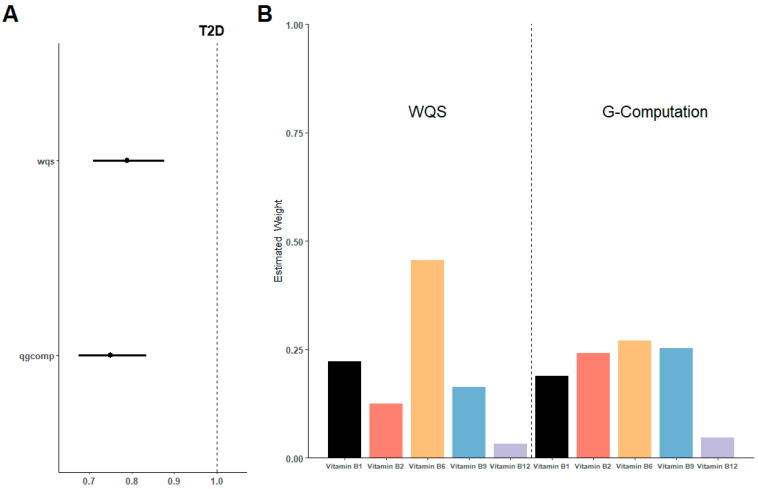
Estimated risk and weighted values of B vitamins for T2D by WQS and QGcomp models. (**A**) Associations of B vitamins with T2D risk. (**B**) Weighted values of B vitamins for T2D. Models were adjusted for sex, age, HbA1C (%), education, smoking status, drinking alcohol status, physical activity, body mass index, energy intake, and family history of diabetes.

As shown in Figure 3A, the WQS index of mixed B vitamins negatively correlated with the risk of T2D (OR 0.80, 95%CI 0.71 to 0.88). The weights of the main vitamins in the WQS models were vitamin B6 (45.58%), vitamin B1 (22.27%), vitamin B9 (16.37%), and vitamin B2 (12.49%) in decreasing order, with the proportion of Vitamin B12 less than 5% (Figure 3B).

In the sensitivity analysis (Figure 3A,B), QGcomp models also revealed a negative association between the overall intake of B vitamins and T2D (OR 0.75, 95%CI 0.68 to 0.83). Similar to the WQS model, vitamin B6 still has the highest contribution, accounting for 26.95% of the total.

**Figure 4:**
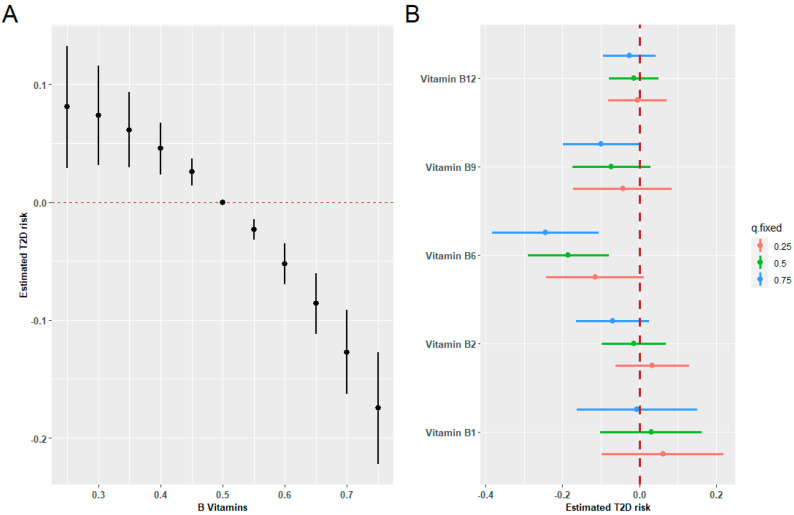
Combined effects of B vitamins on T2D risk estimated by BKMR models. (**A**) The estimated difference in T2D risk and 95%CI when all vitamin levels were held at particular percentiles compared to their medians. (**B**) Associations of a single B vitamin with T2D risk were estimated when other B vitamins were held at their corresponding 25th (red), 50th (green), or 75th (blue) percentile, respectively. Models were adjusted for sex, age, HbA1C (%), education, smoking status, drinking alcohol status, physical activity, body mass index, energy intake, and family history of diabetes.

Figure 4A demonstrates the exposure-response function for the association between mixed B vitamin intakes and T2D risk in BKMR models, indicating a significant negative correlation. Figure 4B illustrates a negative association of vitamin B6 and B9 intakes with incident T2D when other B vitamins were held at high concentrations. No interactions have been observed between the B vitamins. Figure S1 shows the univariate exposure-response functions for each vitamin with the other B vitamins fixed at the median.

### 3.5. Mediation Analyses

**Figure 5:**
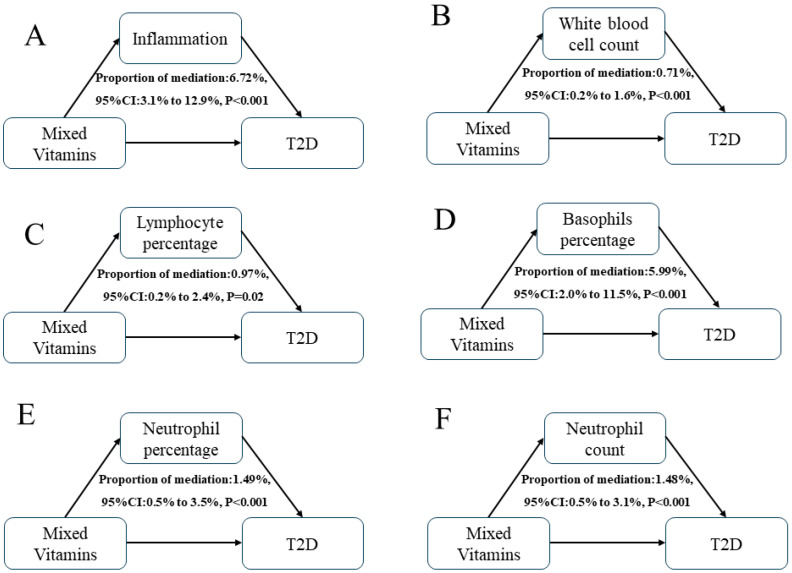
Estimated proportion of the associations between mixed B vitamins and T2D mediated by single (**B**–**F**) or multiple (**A**) inflammatory biomarkers. Models were adjusted for sex, age, HbA1C (%), education, marital status, smoking status, drinking alcohol status, physical activity, body mass index, energy intake, and family history of diabetes. Proportion of mediation (PM)  =  IE/DE  +  IE.

Mediation analyses were performed to assess the potential effect of inflammation on the relationship between mixed B vitamins and T2D (Table 3 and Figure 5). Blood white blood cell count (B), lymphocyte percentage (C), basophils percentage (D), neutrophil percentage (E), and neutrophil count (F) showed a sign mediating role in the associations between mixed B vitamins and T2D risk, with mediation proportions of 0.71%, 0.97%, 5.99%, 1.49%, and 1.48%, respectively. Moreover, a combined mediating model (A) using the joint level of five biomarkers as the mediator exhibited the highest mediation proportion of 6.72% (95%CI 3.1% to 12.9%).

## 4. Discussion

This study found a constant association between joint B vitamin intake and T2D risks in Shanghai residents, with five B vitamins (B1, B2, B6, B9, B12) identified as protective micronutrients for T2D development. Furthermore, vitamin B6 showed the most potent effect on decreasing T2D risks by WQS and QGcomp models. While BKMR models demonstrated a similar negative relationship between B vitamins and the incidence of T2D, no interactions between B vitamins were detected. Additionally, mediation analysis suggested that inflammation might partially account for the inverse association between long-term B vitamin intake and T2D risks.

Regarding the population nutrition status of B vitamins, in comparison to the recommended nutrient intake (RNI) for individuals older than 50 years old in the Chinese Dietary Reference Intakes (2023 edition), the overall intake levels of vitamin B3, B9, and B12 were higher than the recommended levels, while vitamin B1, B2, and B6 intakes were slightly below the recommendations. Additionally, compared to the National Health and Nutrition Examination Survey (NHANES) database, the levels of six B vitamins are similar between the Chinese and US residents [33,34,35]. Consistent with numerous epidemiological studies, our research identified strong links between a single B vitamin intake and incident T2D. Specifically, we uncovered negative associations of T2D risk with vitamin B1 and B2, which agreed with a previous cohort study in Shanghai, China [36]. Furthermore, a meta-analysis found that an increased risk of diabetes was associated with lower levels of various thiamine markers [19]. Furthermore, as a crucial component of one-carbon metabolism, an inverse relationship between dietary folate (vitamin B9) intake and incident T2D was observed, aligning with prior findings [37,38,39,40] in studies such as the Korea Multi-Rural Communities Cohort and the Nurses’ Health Study Ⅱ. Our results revealed a negative correlation between dietary vitamin B12 levels and T2D. A meta-analysis [41] indicated that a deficiency in vitamin B12 was associated with an increased risk of diabetes, with researchers highlighting that higher concentrations of vitamin B12 correlated with lower fasting and postprandial glucose levels [42]. Research on vitamin B6 was relatively scant, but our results suggested that vitamin B6 was associated with a reduced risk of T2D in a Chinese population aged more than 50 years. In animal studies, Okada M. et al. [43] discovered an inverse relationship between vitamin B6 levels and diabetes state. Moreover, a study on fruit flies found [44] that mutations in vitamin-B6-related metabolic genes could lead to diabetes, providing direct evidence that a deficiency in vitamin B6 might contribute to the disease’s onset. However, we found no correlation between vitamin B3 and T2D risks. A previous cross-sectional study conducted in China reported a negative correlation between niacin intake and the risk of developing diabetes [21]. Conversely, a US study found that T2D and obesity were associated with excessive niacin intake [45]. Our current research and the findings from others do not provide conclusive evidence on this association, and further studies are needed to explore the relationship between vitamin B3 and T2D.

Since B vitamins are ubiquitously present in various foods with potential interactions [46], we employed the WQS index to represent the collective effect of mixed B vitamin intake. When facing a high-dimensional and correlated set of exposures, the WQS regression method outperforms traditional regression approaches by effectively addressing issues such as collinearity and variance inflation. Moreover, it demonstrates improved accuracy compared to shrinkage methods like Lasso, Adaptive Lasso, and Elastic Net, making it particularly effective in mixed exposure models [47]. The WQS results showed that the aggregate level of B vitamins was inversely associated with the risk of T2D, consistent with the results from both QGcomp and BKMR models. In particular, within the WQS model, vitamin B6 was identified as the most influential B vitamin on T2D risks, which might be attributed to its myriad metabolic functions. On the one hand, vitamin B6 could affect homocysteine levels and significantly impact tryptophan metabolism, which might further influence insulin activity and secretion [48]. Moreover, a deficiency in vitamin B6 could lead to the accumulation of xanthurenic acid (XA), possessing pathological alterations in the pancreatic beta-cell tissues [49]. On the other hand, decreased availability of vitamin B6 could also affect insulin resistance by increasing adipose tissue and lipogenesis [50,51,52].

In contrast to our observational study, few randomized clinical trials have demonstrated the efficacy of supplementing with B vitamins in reducing the risk of T2D. For instance, one clinical trial indicated that the daily combined administration of folic acid (2.5 mg), vitamin B6 (50 mg), and vitamin B12 (1 mg) had no impact on the risk of T2D among American women with a history of cardiovascular diseases (CVD) or risk factors for CVD [53]. This may be attributed to the fact that RCT studies did not consider the intake of nutrients from the diet, which is a primary source of B vitamins. Also, clinical trials may not be feasible for examining the long-term effects of B vitamin intake on diabetes risk. Lastly, the clinical trial was conducted among older individuals with cardiovascular disease or at risk for cardiovascular disease, who are more susceptible to diabetes [54]. Therefore, supplementing with B vitamins or increasing dietary intake of B vitamins might not benefit this population as much as it appears to benefit ostensibly healthy individuals.

In the dietary structure of the study participants, rice and its products such as rice congee, rice noodles, and rice cakes are staple foods. Although these foods are not particularly rich in vitamins B1, B3, and B6, they still constitute the highest proportion of these vitamins in the diet. Fresh vegetables are an important source of B vitamins for the study residents. They are the second largest source of vitamins B1, B3, and B6 and the primary source of vitamin B9. Meat contains substantial vitamin B6, B1, and B12. Therefore, we recommend that residents consider increasing their intake of fresh vegetables and meat.

Despite the nutritional outcomes regarding complex B vitamin deficiency, inflammation was generally involved [55]. B vitamin deficiency could increase homocysteine concentrations, damaging endothelial cells and vascular walls [56] and leading to a cascade of inflammatory reactions [57]. Also, inflammatory responses that behaved as a critical driving factor in insulin resistance might be causally linked to the onset and progression of T2D [58]. As expected, the simultaneous exposure to the five included biomarkers was positively associated with the incidence of diabetes, as shown in Table S3. Furthermore, dietary interventions with combined B vitamins were more effective in combating inflammation than using a single B vitamin alone [59]. In this research, we hypothesized a mediating effect of inflammation between the joint B vitamin intake and the occurrence of T2D. The combined model showed a mediation proportion of 6.72%, indicating a specific role of blood inflammatory biomarkers. It joined the numerous pieces of evidence confirming inflammatory markers as the indicators of developing T2D and its eventual long-term complications [60].

From a mechanistic perspective, we focused on inflammation. Antidiabetic medications are important factors that influence inflammation, and both metformin and sulfonylureas can affect inflammation [61,62]. In this study, the diagnosis of diabetes took into account the use of antidiabetic medications, and individuals who were using these medications at baseline were excluded. If participants started using antidiabetic medications during follow-up, they were diagnosed with diabetes. Therefore, the study population did not use antidiabetic medications from enrollment until the occurrence of diabetes. Exercise is also an important factor that affects inflammation [63] and diabetic risks [64], and we adjusted for physical activity in both the logistic and WQS models. We also adjusted for physical activity in the mediation model.

This study has several strengths. We employed multiple methodologies to investigate the associations between the single or mixed intake of B vitamins and T2D risks in a sizeable medical-registry-based cohort. Then, various models, including WQS, QGcomp, and BKMR, were used to explore the individual contributions and interactions of B vitamins to the joint effects. Mediation analysis was also conducted to explore potential mechanisms.

However, there are also limitations. Due to the absence of dietary supplement data in the survey, the intake of B vitamins might not accurately reflect the actual amount metabolized in the body. At the same time, we did not have data indicating that these populations alter their dietary patterns in the presence of other diseases. Secondly, the diagnosis of newly onset diabetes was solely based on medical assessments, which could potentially underestimate the number of diabetes cases. This underestimation might be a reason for the observed lower incidence of diabetes within the cohort. Thirdly, we only analyzed the association of six B vitamins with diabetes, leaving the need to study other B-group nutrients. In addition, the blood inflammatory biomarkers we utilized might not fully represent the individual levels of inflammation. We did not have a complete dataset for hemoglobin (Hb) and platelet count and lacked data for hematocrit (Ht) and corpuscular volume (MCV). The absence of these additional biomarkers might limit the comprehensiveness of our findings. Lastly, there might be other uncovered residual and unmeasured confounders that could introduce bias into our analysis. One notable example is that menopause can interfere with metabolic conditions and influence the predisposition to type 2 diabetes. As this factor was not specifically controlled for in our study, it might have influenced the results. Given these shortcomings, we must interpret and generalize our findings with caution. Further studies are needed to support our observations.

## 5. Conclusions

In summary, our findings suggested that the intake of B vitamins (B1, B2, B6, B9, B12) was inversely associated with incident T2D in Shanghai residents. The mixture-exposed analyses consistently showed the negative correlations between dietary B vitamins and a decreased T2D risk, primarily driven by vitamin B6. Residents could consider increasing their intake of fresh vegetables and meat. Furthermore, we observed that the association between B vitamins and the risk of T2D might be partially mediated by inflammation. These insights highlighted B vitamins as dietary protective nutrients against T2D and suggested that the inflammatory responses could be an underlying mechanism, offering guidance for future diabetes prevention strategies. To validate and expand our findings, future research could investigate the relationship between B vitamins and inflammatory markers to further elucidate the mediating role in the protective effects of B vitamins against T2D. Additionally, similar studies could be conducted in different regions and cultural contexts to determine the generalizability of our findings, aiding in the development of global dietary recommendations.

## Figures and Tables

**Table 1:** Basic characteristics of participants by T2DM among adults, SSACB 2016–2022.

Characteristics	Non-T2DM (*n* = 43,121)	T2DM (*n* = 1839)	*p* Value
Age	55.91 (11.73)	59.42 (9.01)	<0.001
Gender			0.269
Male	16,957 (39.3)	699 (38.0)	
Female	26,164 (60.7)	1140 (62.0)	
BMI (kg/m^2^)	24.04 (4.24)	25.00 (3.48)	<0.001
HbA1c (%)	5.58 (0.40)	5.80 (0.41)	<0.001
Total energy intake (kcal/d)	1987.03 (654.74)	1955.19 (644.97)	0.041
Family history of diabetes			<0.001
Yes	5147 (11.9)	297 (16.2)	
No	37,974 (88.1)	1542 (83.8)	
Smoking			0.276
Yes	9351 (21.7)	419 (22.8)	
Never	33,770 (78.3)	1420 (77.2)	
Drinking alcohol			0.014
Yes	5281 (12.2)	261 (14.2)	
Never	37,840 (87.8)	1578 (85.8)	
Physical activity			0.030
Low	6244 (14.5)	226 (12.3)	
Medium	16,907 (39.2)	732 (39.8)	
High	19,970 (46.3)	881 (47.9)	
Educational level			<0.001
Primary school or below	14,918 (34.6)	774 (42.1)	
Junior high school	21,299 (49.4)	917 (49.9)	
Senior high school or above	6904 (16.0)	148 (8.0)	
Thiamine (mg/d)	0.90 (0.34)	0.87 (0.33)	<0.001
Riboflavin (mg/d)	0.89 (0.44)	0.84 (0.39)	<0.001
Niacin (md/d)	14.97 (5.98)	14.57 (5.76)	0.005
Pyridoxine (mg/d)	1.25 (0.46)	1.19 (0.42)	<0.001
Cobalamin (mcg/d)	4.03 (2.89)	3.69 (2.6)	<0.001
Folate (mcg/d)	801.03 (403.20)	786.16 (414.70)	0.122

Continuous variables are presented as mean ± SE. Categorical variables were presented as *n* (%). *SSACB* The Shanghai Suburban Adult Cohort and Biobank. *BMI* body mass index, *HbA1C* glycated hemoglobin, *SE* standard error, *n* numbers of subjects, *%* weighted percentage.

**Table 2:** Dietary sources and contribution of B vitamins among the participants (%).

Food Name	Vitamin B1	Vitamin B2	Vitamin B3	Vitamin B6	Vitamin B9	Vitamin B12
Edible salt	0.00	0.00	0.00	0.00	0.00	0.00
Sugar	0.09	0.13	0.06	0.00	0.00	0.00
Soy sauce	0.70	1.90	1.40	0.58	0.23	0.00
Edible oils	0.39	1.69	0.35	6.47	0.00	1.21
Rice and rice-based products	36.02	10.94	32.17	27.52	20.69	0.00
Wheat flour and wheat-flour-based products	8.39	2.98	4.45	1.25	15.99	0.00
Coarse grains and their products	4.01	2.2	2.27	3.86	3.16	0.17
Potatoes and their products	1.89	0.56	1.13	3.03	0.69	0.00
Fresh vegetables	9.54	14.58	17.50	21.78	29.04	0.00
Mushrooms	4.76	6.93	3.26	2.28	1.57	0.00
Fresh fruits	5.31	6.36	3.75	4.67	1.66	10.07
Fresh milk and cheese products	1.45	6.90	0.27	0.52	0.34	5.00
Milk powder	0.01	0.03	0.00	0.01	0.00	0.02
Yogurt	1.37	5.16	0.28	0.66	0.29	2.81
Pork	11.54	5.42	9.43	5.70	0.00	5.30
Other livestock meats	0.51	1.41	3.00	2.71	0.10	3.04
Poultry	0.75	3.47	4.41	1.18	0.06	5.56
Animal offal	0.72	5.24	1.88	2.19	1.63	0.00
Freshwater fish	0.67	2.29	2.96	1.62	0.28	7.28
Saltwater fish	0.35	7.59	1.57	0.98	0.19	14.99
Shrimp, crab, and mollusk	0.44	0.62	2.68	0.61	0.14	24.37
Soy milk	0.50	0.46	0.23	1.18	0.52	0.00
Tofu and soy-based products	1.35	0.90	0.45	1.44	11.60	0.00
Eggs	3.20	7.09	0.41	0.87	8.46	8.59
Nuts	2.47	1.46	1.81	4.63	2.46	0.00
Carbonated beverages	0.00	0.00	0.00	0.00	0.00	0.00
Pure fruit and vegetable juices	0.00	0.00	0.00	0.00	0.01	0.00
Other sugary beverages	0.11	0.10	0.35	0.08	0.05	0.00
Candies and chocolates	0.16	0.30	0.1	0.06	0.08	0.00
Deep-fried pasta	0.33	0.23	0.53	0.24	0.13	0.35
Pickled and salted foods	0.78	1.08	0.77	1.34	0.29	0.00
Processed meat products	1.09	0.68	0.99	1.86	0.01	4.21
Baked goods	1.12	1.29	1.54	0.68	0.32	7.03

**Table 3:** Blood biomarkers of participants by T2D and the mediated proportion in mediation analyses.

Biomarker	Non-T2D (*n* = 43,121)	T2D (*n* = 1839)	PM (%)	*p*-Value
White blood cell count	5.92 (1.52)	6.09 (1.57)	0.7120	<0.001
Monocyte percentage	6.41 (1.68)	6.43 (1.62)	0.4579	0.28
Monocyte count	0.38 (0.13)	0.39 (0.13)	−0.3130	0.16
Lymphocyte percentage	33.74 (7.83)	33.45 (7.92)	0.9696	0.02
Lymphocyte count	1.97 (0.60)	2.01 (0.60)	−0.0580	0.56
Urine creatinine	12,671.04 (7043.03)	12,150.34 (6574.80)	1.4976	0.08
Basophil percentage	0.51 (0.32)	0.49 (0.29)	5.9917	<0.001
Basophil count	0.14 (0.87)	0.14 (0.85)	0.4175	0.56
Eosinophil percentage	2.07 (1.77)	2.07 (1.65)	0.1188	0.55
Eosinophil count	0.38 (1.29)	0.39 (1.31)	−0.3430	0.80
Serum albumin/globulin ratio	1.84 (0.40)	1.82 (0.33)	−0.4210	0.72
Serum creatinine	70.55(19.53)	69.32 (15.69)	−0.1000	0.84
Neutrophil percentage	57.27 (8.32)	57.55 (8.39)	1.4926	<0.001
Neutrophil count	3.42 (1.16)	3.54 (1.21)	1.4802	<0.001
AST/ALT	1.29 (0.44)	1.23 (0.43)	0.1955	0.94

Variables are presented as mean ± SE. Models were adjusted for sex, age, education, smoking status, drinking alcohol status, physical activity, body mass index, energy intake, and family history of diabetes.

## Data Availability

The data presented in this study are available on request from the corresponding author. The data are not publicly available due to privacy.

## Supplementary Materials

The following supporting information can be downloaded at: https://www.mdpi.com/article/10.3390/nu16121901/s1, Table S1: Associations of single B vitamin intake with T2D risks; Table S2: Associations of single B vitamin intake with T2D risks; Table S3: OR (95%CI) in T2D associated with blood biomarkers by QGcomp model; Figure S1: Univariate exposure-response functions for each vitamin with the other B vitamins fixed at the median.
