# Supplementary material for: Joint B Vitamin Intake and Type 2 Diabetes Risk: The Mediating Role of Inflammation in a Prospective Shanghai Cohort
Source: Nutrients. 2024 Jun 16;16(12):1901. doi: 10.3390/nu16121901 (PMC11206684; doi:10.3390/nu16121901)
Supplement: Supplementary file 1 [file nutrients-16-01901-s001.zip › nutrients-3020725-supplementary.pdf]

Table S1. Associations of single B vitamin intake with T2D risks.

Vitamins	Continuous OR (95% CI)	Q1	Q2 OR (95% CI)	Q3 OR (95% CI)	Q4 OR (95% CI)	*p* for trend
Thiamine (B1)	0.83 (0.74,0.92)	1.00 (reference)	0.94 (0.82,1.09)	0.76 (0.64,0.91)	0.65 (0.51,0.83)	<0.001
Riboflavin(B2)	0.88 (0.82,0.95)	1.00 (reference)	1.11 (0.98,1.27)	0.97 (0.84,1.12)	0.84 (0.71,0.99)	0.0193
Niacin (B3)	0.97 (0.90,1.04)	1.00 (reference)	0.95 (0.82,1.09)	0.92 (0.78,1.08)	0.89 (0.73, 1.09)	0.25
Pyridoxine (B6)	0.75 (0.67,0.84)	1.00 (reference)	0.96 (0.83,1.11)	0.82 (0.69,0.98)	0.65 (0.52,0.82)	<0.001
Folate (B9)	0.94 (0.86,1.03)	1.00 (reference)	0.85 (0.74,0.98)	0.85 (0.73,0.99)	0.77 (0.62,0.94)	0.016
Cobalamin (B12)	0.91 (0.86,0.96)	1.00 (reference)	0.98 (0.86,1.11)	0.94 (0.82,1.08)	0.80 (0.69,0.92)	0.003

Logistic regression models were adjusted for sex, age, HbA1C (%), education, smoking status, drinking alcohol status, physical activity, body mass index, energy intake and family history of diabetes.

Table S2. Associations of single B vitamin intake with T2D risks.

Vitamins	Continuous OR (95% CI)	Q1	Q2 OR (95% CI)	Q3 OR (95% CI)	Q4 OR (95% CI)	p for trend
Thiamine(B1)	0.85 (0.76,0.94)	1.00 (reference)	0.95 (0.82,1.09)	0.77 (0.65,0.92)	0.68 (0.54,0.87)	<0.001
Riboflavin(B2)	0.92 (0.86,0.98)	1.00 (reference)	1.14 (1.00,1.30)	1.02 (0.89,1.18)	0.92 (0.78,1.08)	0.202
Niacin(B3)	0.99 (0.92,1.06)	1.00 (reference)	0.95 (0.83,1.10)	0.94 (0.80,1.10)	0.94 (0.77, 1.14)	0.52
Pyridoxine(B6)	0.77 (0.69,0.86)	1.00 (reference)	0.96 (0.84,1.11)	0.84 (0.71,0.99)	0.68 (0.54,0.85)	<0.001
Folate(B9)	0.90 (0.82,0.99)	1.00 (reference)	0.83 (0.72,0.95)	0.81 (0.70,0.95)	0.71 (0.58,0.88)	0.001
Cobalamin(B12)	0.91 (0.86,0.97)	1.00 (reference)	1.00 (0.88,1.14)	0.98 (0.86,1.12)	0.85 (0.74,0.98)	0.039

Logistic regression models were adjusted for sex, age, education, smoking status, drinking alcohol status, physical activity, body mass index, energy intake and family history of diabetes.

Table S3. OR (95%CI) in T2D associated with blood biomarkers by QGcomp model.

	OR	Lower CI	Upper CI	P
Biomarkers	1.086	1.037	1.136	< 0.001

Models were adjusted for sex, age, education, smoking status, drinking alcohol status, physical activity, body mass index, energy intake and family history of diabetes.

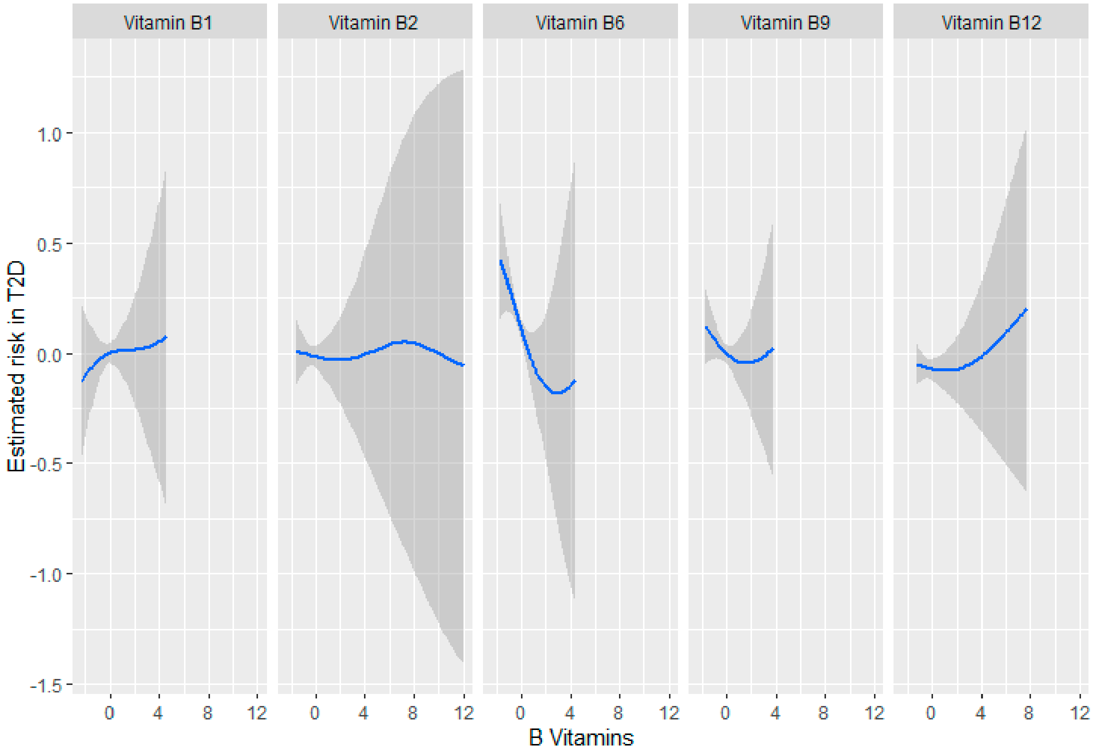

Figure S1. Univariate exposure-response functions for each vitamin with the other B vitamins fixed at the median. The results were assessed by the Bayesian Kernel Machine Regression (BKMR) models. Models were adjusted for sex, age, HbA1C (%), education, marital status, smoking status, drinking alcohol status, physical activity, body mass index, energy intake, and family history of diabetes.
